# Responsible use of population neuroscience data: Towards standards of accountability and integrity

**DOI:** 10.1016/j.dcn.2024.101466

**Published:** 2024-10-18

**Authors:** Sandra A. Brown, Hugh Garavan, Terry L. Jernigan, Susan F. Tapert, Rebekah S. Huber, Daniel Lopez, Traci Murray, Gayathri Dowling, Elizabeth A. Hoffman, Lucina Q. Uddin

**Affiliations:** Departments of Psychology and Psychiatry, University of California San Diego, San Diego, CA, United States; Department of Psychiatry, University of Vermont, 1 South Prospect UHC 6436, Burlington, VT 05401, United States; Departments of Cognitive Science, Psychiatry, and Radiology, University of California San Diego, San DiegoCA 92093, United States; Department of Psychiatry, University of California, San Diego, San Diego, CA, United States; Department of Psychiatry, Oregon Health & Science University, 3181 SW Sam Jackson Park Rd, UHN-80R1, Portland, OR 97239, United States; Division of Extramural Research, National Institute on Drug Abuse, Bethesda, MD 20877, United States; Department of Psychiatry and Biobehavioral Sciences, Semel Institute for Neuroscience and Human Behavior, University of California, 760 Westwood Plaza, Los Angeles, CA 90024, United States

**Keywords:** Responsible data use, Population neuroscience, Health equity, Inclusivity, Race, Genetic, Ancestry, Population descriptors

## Abstract

This editorial focuses on the issue of data misuse which is increasingly evidenced in social media as well as some premiere scientific journals. This issue is of critical importance to open science projects in general, and ABCD in particular, given the broad array of biological, behavioral and environmental information collected on this American sample of 12.000 youth and parents. ABCD data are already widely used with over 1000 publications and twice as many citations per year as expected (relative citation index based on year, field and journal). However, the adverse consequences of misuse of data, and inaccurate interpretation of emergent findings from this precedent setting study may have profound impact on disadvantaged populations and perpetuate biases and societal injustices.

For over a decade the open science movement has been heralded for accelerating scientific discovery resulting in more rapid translation of research findings that are critical to public health (McKiernan et. al., 2016). Open science efforts have led to the availability of large population neuroscience datasets that have revolutionized cognitive and clinical neuroscience (Uddin et al., 2024, Biswal et al., 2010), especially with respect to health disparities research that is critical for informing the development of interventions to promote health equity (Harnett et al., 2024). Such big data use comes with the challenge and responsibility to ensure ethical conduct of data analysis and interpretation to prevent further stigmatization of historically marginalized groups (Laird, 2021). As investigators, federal partners, and journal editors, we endeavor to communicate expectations surrounding acceptable data use and discourage misuse of population neuroscience data.

Complex datasets like the Adolescent Brain Cognitive Development^SM^ (ABCD) Study underscore the need for caution in how human subjects' data are generated, modeled, analyzed, interpreted, and communicated to both fellow scientists and the community at large. Creators and users of large datasets seek to promote data use that avoids harm to individuals or populations. Using the ABCD Study as an example, we provide recommendations to help enable large datasets to be used responsibly and to not further stigmatize, marginalize, or otherwise harm minoritized groups.

The primary research goal of the ABCD Study^(R)^ is to determine how experiences during childhood interact to influence brain and cognitive development including social, behavioral, and health outcomes (Jernigan et al., 2018). ABCD Study data collection and sharing are collaborative efforts requiring expertise from multiple scientific disciplines, and academic, industry, and government institutions, as well as the communities under study. The ABCD dataset has the potential to produce valuable insights into cultural and environmental factors relevant to youth that may influence different health trajectories. However, when studying health disparities, researchers often broadly define social constructs such as race, ethnicity, gender, and socioeconomic status, using them as proxies for other experiences. Given the complexity of these constructs, which are often multidimensional, use as independent variables in isolation is to be avoided (Hoffman et al., 2022). To address this common challenge, it is recommended that models incorporate contextualizing variables such as family, social and neighborhood environments, school and recreational experiences, vocational environment, acculturation, and perceived discrimination, especially in predictive models that attempt to explain variability in behavior, cognition, and mental and physical health outcomes. The use of these constructs necessitates thoughtful consideration to prevent further stigmatizing or marginalizing youth. By way of example, attributions of differences in cognitive measures between children of different reported races are particularly sensitive. Interpretations may be incomplete and misleading when results do not consider and contextualize findings in both the communities from which the samples were drawn and historical context. With these additional considerations, authors can enhance the accuracy of scientific understanding and avoid the current and legacy of discrimination-induced stress and marginalization that may contribute to or be the cause of those differences.

Fortunately, several recent papers have been published that can empower big data users to make discoveries without inadvertently stigmatizing portions of the population under study (e.g., Ford et al., 2023). Researchers are encouraged to:

• Consider the broader social context in which development occurs and the potential for developmental change and adaptation to the environment (Simmons et al., 2021).
• Employ theory-based and community-informed approaches to determine relevant variables for a given research question (Cardenas-Iniguez et al., 2024).
• Be aware of the assumptions made and potential limitations when choosing variables to measure social constructs (Hoffman et al., 2022).
• Avoid misrepresenting constructs as proxies for social and environmental forces (e.g., race as a proxy for racism) and instead directly measure critical social, cultural and environmental variables (Cardenas-Iniguez and Gonzalez, 2024, Hoffman et al., 2022).
• Use recommended practices for selecting and using population descriptors (e.g., race, ethnicity, and geographic origin) in genetics and genomics research (e.g., National Academies of Sciences, Engineering, and Medicine, 2023 reports; Bird and Carlson, 2024; Feero et al., 2024; Cardenas-Iniguez and Gonzalez, 2024).
• Include a justification for the use of race and ethnicity variables along with information gathered from the community that the study represents (Cardenas-Iniguez and Gonzalez, 2024).
• Utilize recommended practices for reproducible research, analytical procedures and reporting of results in ABCD projects (Lopez et al., 2024).
• Engage relevant communities in the design, interpretation, and communication of findings (Isreal et al., 2024, Gibbons and Pérez-Stable, 2024).
• Provide a thoughtful interpretation and discussion of findings with a clear description of limitations, to avoid possible negative interpretations or misinterpretation of the findings by readers (Saragosa-Harris et al., 2022).

Professional reviews and published reports include recommendations supporting community-engagement to enhance the validity of constructs and measures, and improve the integrity of analytic efforts as well as accuracy of communicated results. While challenging, resources are available to assist in this effort. For example, the principles of Indigenous Data Sovereignty and Governance can guide use of public datasets for research related to American Indian and Alaska Native populations (White et al., 2023). Recommendations are available to assist at the project design, development and analytic stages, to enhance equity in questions and analytic processes (Bodison et al., 2023), such as “Have you acknowledged any potential bias in measures/constructs (known or suspected)?” and “Have you been careful to contextualize variables, such as race/ethnicity/, gender, and/or SES?”.

The NIH has taken steps to ensure that researchers accessing the ABCD dataset are properly trained in responsible use of data. For upcoming data releases, training modules accompany the Data Use Certification (DUC) workflow and must be completed before data access is granted. For 2024 data releases and beyond, the DUC includes language about compliance with human subjects’ protection requirements. In particular, researchers must agree to conform to the ethical conduct of research and consider any potential psychological, social, economic, and other potentially harmful impacts their research results could have on individuals, communities, and society, and take steps to minimize such impacts. Further to assist investigators, the ABCD Data Analysis, Informatics, and Resource Center and Justice, Equity Diversity and Inclusion (JEDI) Workgroups have articulated special considerations for the use of variables, particularly historically misused social constructs. The ABCD Wiki includes specific notes for use of individual variables and composite metrics, and recommended practices to aid in consideration of confounding and explanatory influences in effects under examination. These transparency efforts can improve the reproducibility of ABCD findings (Lopez et al., 2024).

With increasing availability of large, multidimensional datasets, researchers bear additional responsibility for ethical measure development, data use, analysis, interpretation, and communication of findings. While new strategies and techniques will continue to be developed, it is already the responsibility of the individual researcher to attend to best practice data use methods to minimize perpetuation of stigma and harm to individuals, communities, and society. We strongly encourage researchers to consider the recommendations provided and elsewhere. We acknowledge these methods may present challenges and may not be optimal in every context, however, striving towards these standards is crucial to expand knowledge of human development, and ensure fair, inclusive, and just science.

## CRediT authorship contribution statement

**Traci Murray:** Writing – review & editing, Writing – original draft, Resources, Project administration. **Daniel Lopez:** Writing – review & editing, Writing – original draft, Project administration, Conceptualization. **Rebekah S. Huber:** Writing – review & editing, Writing – original draft, Project administration, Conceptualization. **Susan F. Tapert:** Writing – review & editing, Conceptualization. **Terry L. Jernigan:** Writing – review & editing, Supervision, Conceptualization. **Hugh Garavan:** Writing – review & editing. **Sandra A. Brown:** Writing – review & editing, Writing – original draft, Supervision, Resources, Project administration. **Lucina Q. Uddin:** Writing – review & editing, Writing – original draft, Supervision, Resources, Project administration, Conceptualization. **Elizabeth A. Hoffman:** Writing – review & editing, Writing – original draft, Resources, Project administration, Conceptualization. **Gayathri Dowling:** Writing – review & editing, Resources, Conceptualization.

## Declaration of Competing Interest

The authors declare that they have no known competing financial interests or personal relationships that could have appeared to influence the work reported in this paper.

## References

[bib1] Bird Kevin A., Carlson Jedidiah (2024). TypologicaL Thinking In Human Genomics Research Contributes To The Production And Prominence Of Scientific Racism. Front. Genet..

[bib2] Biswal Bharat B., Mennes Maarten, Zuo Xi-Nian, Gohel Suril, Kelly Clare, Smith Steve M., Beckmann Christian F. (2010). Toward discovery science of human brain function. Proc. Natl. Acad. Sci. USA.

[bib3] Bodison, S.C., Nagel, B., Lopez, D., Huber, R., WG3, M. of A. J. (2023). Equity-Focused Questions for Researchers using the ABCD Study. ttps://doi.org/10.17605/OSF.IO/PM7SY.

[bib4] Cardenas-Iniguez Carlos, Gonzalez Marybel Robledo (2024). Recommendations for the responsible use and communication of race and ethnicity in neuroimaging research. Nat. Neurosci..

[bib5] Cardenas-Iniguez Carlos, Schachner Jared N., Ip Ka.I., Schertz Kathryn E., Gonzalez Marybel R., Abad Shermaine, Herting Megan M. (2024). Building towards an adolescent neural urbanome: expanding environmental measures using linked external data (LED) in the ABCD study. Dev. Cogn. Neurosci..

[bib6] Feero W.Gregory, Steiner Robert D., Slavotinek Anne, Faial Tiago, Bamshad Michael J., Austin Jehannine, Korf Bruce R., Flanagin Annette, Bibbins-Domingo Kirsten (2024). Guidance on use of race, ethnicity, and geographic origin as proxies for genetic ancestry groups in biomedical publications. JAMA J. Am. Med. Assoc..

[bib7] Ford C.A., Boyer C.B., Halpern C.T., Katzman D.K., Ross D.A. (2023). The Journal of Adolescent Health’s commitment to diversity, equity, and inclusion. J. Adolesc. Health.

[bib8] Gibbons G.H., Pérez-Stable E.J. (2024). Harnessing the power of community-engaged research. Am. J. Public Health.

[bib9] Harnett N.G., Merrill L.C., Fani N. (2024). Racial and ethnic socioenvironmental inequity and neuroimaging in psychiatry: a brief review of the past and recommendations for the future. Neuropsychopharmacol..

[bib10] Hoffman Elizabeth A., LeBlanc Kimberly, Weiss Susan R.B., Dowling Gayathri J. (2022). Transforming the future of adolescent health: opportunities from the adolescent brain cognitive development study. J. Adolesc. Health.: Off. Publ. Soc. Adolesc. Med..

[bib11] Isreal B.A., Schulz A.J., Parker E.A., Becker A.B. (2024). Community-based participatory research: policy recommendations for promoting a partnership approach in health research. Am. J. Public Health.

[bib12] Jernigan Terry L., Brown Sandra A., Dowling Gayathri J. (2018). The adolescent brain cognitive development study. J. Res. Adolesc. Off. J. Soc. Res. Adolesc..

[bib13] Laird Angela R. (2021). Large, open datasets for human connectomics research: considerations for reproducible and responsible data use. NeuroImage.

[bib14] Lopez D.A., Cardenas-Iniguez C., Subramaniam P., Adise S., Bottenhorn K.L., Badilla P., Mukwekwerere E., Tally L., Ahanmisi O., Bedichek I.L., Matera S.D., Perez-Tamayo G.M., Sissons N., Winters O., Harkness A., Nakiyingi E., Encizo J., Xiang Z., Wilson I.G., Smith A.N., Huber R.S. (2024). Transparency and reproducibility in the ADolescent Brain Cognitive Development (ABCD) study. Dev. Cogn. Neurosci..

[bib15] McKiernan E.C., Bourne P.E., Brown C.T., Buck S., Kenall A., Lin J., Yarkoni T. (2016). How open science helps researchers succeed. elife.

[bib16] National Academies of Sciences, Engineering, and Medicine (2023).

[bib17] Saragosa-Harris N.M., Chaku N., MacSweeney N., Guazzelli W., Scheuplein M., Feola B. (2022). A practical guide for researchers and reviewers using the ABCD Study and other large longitudinal datasets. Dev. Cogn. Neurosci..

[bib18] Simmons Cortney, Conley May I., Gee Dylan G., Baskin-Sommers Arielle, Barch Deanna M., Hoffman Elizabeth A., Huber Rebekah S. (2021). Responsible use of open-access developmental data: the adolescent brain cognitive development (ABCD) study. Psychol. Sci..

[bib19] Uddin, Lucina Q., F. Xavier Castellanos, Vinod Menon. 2024. Resting state functional brain connectivity in child and adolescent psychiatry: where are we now? Neuropsychopharmacol.: Off. Publ. Am. Coll. Neuropsychopharmacol. 10.1038/s41386-024-01888-1. PMC11525794 38778158

[bib20] White Evan J., Mara J.Demuth, Wiglesworth Andrea, Coser Ashleigh D., Garrett Brady A., Kominsky Terrence K., Jernigan Valarie, Thompson Wesley K., Paulus Martin, Aupperle Robin (2023). Five recommendations for using large-scale publicly available data to advance health among american indian peoples: the adolescent brain and cognitive development (ABCD) studySM as an illustrative case. Neuropsychopharmacol. Off. Publ. Am. Coll. Neuropsychopharmacol..

